# On the interplay between speech perception and production: insights from research and theories

**DOI:** 10.3389/fnins.2024.1347614

**Published:** 2024-01-25

**Authors:** Meisam K. Arjmandi, Roozbeh Behroozmand

**Affiliations:** ^1^Translational Auditory Neuroscience Lab, Department of Communication Sciences and Disorders, Arnold School of Public Health, University of South Carolina, Columbia, SC, United States; ^2^Speech Neuroscience Lab, Department of Speech, Language, and Hearing, Callier Center for Communication Disorders, School of Behavioral and Brain Sciences, The University of Texas at Dallas, Richardson, TX, United States

**Keywords:** speech perception-production, auditory-motor integration, perception-driven adaptation, real-time auditory feedback, impaired peripheral auditory processing

## Abstract

The study of spoken communication has long been entrenched in a debate surrounding the interdependence of speech production and perception. This mini review summarizes findings from prior studies to elucidate the reciprocal relationships between speech production and perception. We also discuss key theoretical perspectives relevant to speech perception-production loop, including hyper-articulation and hypo-articulation (H&H) theory, speech motor theory, direct realism theory, articulatory phonology, the Directions into Velocities of Articulators (DIVA) and Gradient Order DIVA (GODIVA) models, and predictive coding. Building on prior findings, we propose a revised auditory-motor integration model of speech and provide insights for future research in speech perception and production, focusing on the effects of impaired peripheral auditory systems.

## Introduction

Debates on whether spoken communication involves both sides of speech/language production and perception/comprehension has shaped theories and research in the field. One side argues for a “*general auditory*” view, stating that speech perception involves processing acoustic signals independent of production components (Kluender, 1994; Diehl et al., 2004), despite substantial evidence supporting the contrary perspective (Casserly and Pisoni, 2010). This dichotomy echoes broader debates in cognitive psychology, where the idea of separate modules for perception and action, often called a “*cognitive sandwich*,” has been contested (Hurley, 2008). The same conclusion applies broadly to language production and comprehension as an important form of cognitive processing, involving both perception and action. These two components need to work in tango to establish “*signal parity*” between the produced and perceived representations for successful bidirectional communication (Liberman and Mattingly, 1989; Massaro, 2014). The entire system of speech production and perception forms a dynamic process with two cooperating sides to construct a stable and effective system of “*speech chain*” for effective verbal communication (Denes and Pinson, 1963).

Human language development is thought to either begin with inherent linguistic abilities (e.g., Chomsky’s universal grammar theory; Chomsky, 1995) or with no innate knowledge, i.e., a blank slate (e.g., Skinner’s behaviorist theory; Skinner, 1938). These ideas form competing theories that are still being debated. The factor of development in both perception and production of language requires an interwoven interaction between these two systems (Kuhl et al., 2008; Turnball and Justice, 2017). The strong linkage between perceptual representation of speech sounds and the degree of exposure to language and vocal imitation has been demonstrated by native language magnet expanded (NLM-e) model of speech perception (Kuhl et al., 2008), as well as the significant impacts of early language input on speech and language outcomes (Hart and Risley, 1995; Weisleder and Fernald, 2013; Arjmandi et al., 2022), demonstrating this connection during developmental stages. As stated by NLM-e model, organizing the phonetic perceptual space into prototypes space during development allows children to form a perceptual map for representation of linguistic phonetic units (Kuhl et al., 2008). These perceptual maps of speech are later used by children to produce sounds and words of their native language (Kuhl et al., 2008), demonstrating the tight interaction between the encoding and decoding pathways for the translation of speech to language and vice versa.

## Connection between speech perception and production

The closest process to perceiving speech is its representation in action, namely speech production. Early support for a link between speech perception and production comes from Gregory and Webster (1996), showing convergence in speech patterns between speakers of different status in dyadic interviews. This study provided evidence on how the perception of speech influences the intonational structure of a speaker’s speech through accommodation. Pardo (2006) showed that speakers’ speaking style gradually aligns phonetically (i.e., “*phonetic convergence*”) during a “*real-time*” communication task, especially while playing a communication game (i.e., “*map task*”). Speakers also actively monitor their environment to compensate for any speech quality reduction delivered to listeners. Factors such as ambient background noise, complex multi-talker situations, and the speaker’s psychological state may impact the clarity of the speakers’ speech (Lindblom, 1990). The same compensatory reaction is evident in “*Lombard Speech*” effect where speakers utilize louder or highly articulated speech in noisy conditions to establish a successful communication of linguistic messages (Winkworth and Davis, 1997). Such perception-driven adaptations provide compelling evidence in support of the role of speech perception in production and their interconnection.

## Connection between language comprehension and production

In language, the interconnection between comprehension and production is fundamental to effective communication. Studies on single word naming (Bock, 1994) and sentence completion (Bock and Miller, 1991) have demonstrated the connection between language production and comprehension. The single word naming task, often misunderstood as a purely comprehension-based activity, involves both comprehension and production aspects. This task necessitates the active generation and articulation of words, extending beyond mere comprehension. Sentence completion, which is assumed to be a production task, is also not feasible without comprehension. The connection between comprehension of linguistic units and production of speech is supported by neurobiological evidence as well. One such evidence has been provided by the discovery of “*mirror neurons*” comprising neuronal assemblies in the prefrontal cortex and other areas implicated in speech processing (e.g., Broca’s area) (Hickok, 2011). Shared neuronal activation in speech production and silent listening reinforces the link between speech perception and production, providing neurobiological support for Levelt’s proposed internal feedback loop (Levelt et al., 1999) and Moor’s PRESENCE model (Moore, 2007). These models explain emulation of the articulatory-to-acoustic mapping by speakers or listeners mirroring another speaker’s articulatory map (Levelt et al., 1999; Moore, 2007).

Other neurobiological evidence linking perception and production comes from Fadiga et al. (2002) and Watkins and Paus (2004), who showed that listening to speech, but not non-speech, triggers activity in cortical motor regions, such as Broca’s area, associated with speech articulation such as tongue movement. Hickok et al. (2009), however, argued against the role of mirror neurons in speech perception, underscoring evidence such as the lack of influence on speech comprehension when areas related to speech production, e.g., Broca’s, are impaired. They also highlighted infants’ ability to categorize speech sounds (*categorical perception*) before language production begins as additional evidence for the lack of a direct connection between speech perception and production. Several other studies delineated highly overlapping neural pathways implicated in processing and producing language, providing neurobiological evidence of interconnection between language comprehension and production (Scott and Johnsrude, 2003; Wilson et al., 2004; Chang et al., 2015). A speech perception related rate-dependent neural activation was reported during whispering while speech was not audible (Paus, 1996). In addition, the direct relationship between lip muscle activity and the level of neural activities in Broca’s area suggests that auditory input modulates excitability of motor system during speech perception (Fadiga et al., 2002). Active interaction between speech perception and production systems is further supported by demonstrating the involvement of the same cortical regions in semantic, lexical, and syntactic processing during tasks involving speaking and listening (Menenti et al., 2011, see McGettigan and Tremblay, 2017 for a detailed review).

## Real-time auditory feedback and speech perception-production loop

Experimental data from human (Purcell and Munhall, 2006a,b; MacDonald et al., 2011; Khoshhal Mollasaraei and Behroozmand, 2023) and animal (Eliades and Wang, 2005; Eliades and Wang, 2008; Eliades and Tsunada, 2018) studies highlighting the impacts of the internal and external auditory feedback mechanisms on speech production further underscores the link between perception and production. The internal feedback serves as a self-calibrating mechanism that utilizes an internal model to predict the sensory (e.g., auditory) consequences of intended productions based on previously learned associations between motor commands and their feedback signals. This mechanism allows speakers to adjust phonatory and articulatory movements before and shortly after the onset of speech production without relying on the external auditory feedback, as evident through a centering behavior observed in the early stages of vowel production (Hockett, 1967; Gracco and Abbs, 1987; Niziolek et al., 2013). On the other hand, external feedback allows speakers to maintain phonation and articulation accuracy post-production, update the internal model due to production errors, and drive adaptive behavior for speech motor learning. The auditory feedback is incorporated in some computational models of speech acquisition and production (e.g., DIVA and GODVA models) to facilitate the training of the speech production process and simulate fine-grained articulatory movements (Tourville and Guenther, 2013). Other computational models have also been proposed where auditory perception was defined as a pathway to develop the learning process for speaking (Plant and Kello, 2013). In this context, the connectionist models assume that production and comprehension occur through the same network of nodes and connections such that the same pathway that is used for auditory feedback during production is recruited by individuals to perceive speech of others (MacKay, 1982; Dell, 1988). The artificial perturbation of real-time auditory feedback triggers compensatory motor behaviors that aim to minimize feedback error via modifying phonatory and articulatory movements to match the acoustic characteristics of the intended productions. Real-time shifts in vowel first and second formant frequencies prompt speakers to employ a compensatory strategy and adjust the perturbed formant while leaving the unperturbed formant unchanged (Purcell and Munhall, 2006a,b; MacDonald et al., 2011; Khoshhal Mollasaraei and Behroozmand, 2023). A similar compensatory vocal response has been extensively demonstrated in response to pitch-shifted auditory feedback (Behroozmand and Larson, 2011; Behroozmand et al., 2016). This well-known phenomenon of “*sensorimotor adaptation*” demonstrates the critical role of speech perception-production loop for real-time self-monitoring of speech output (Houde and Jordan, 2002; Villacorta et al., 2007).

## Speech perception-production in theories

The interconnection between speech perception and production is a fundamental aspect in most theories of speech perception or speech production. The “*hyper-articulation and hypo-articulation*” (H&H) theory is one of the earliest theories of speech production which aligns with the notion of perception-driven adaptation in speech production. H&H theory highlights the influence of listeners and environmental factors on speakers’ adaptive behavior, wherein they adjust their articulatory patterns to balance between saving effort and making their communication clear (Lindblom, 1990).

Speakers incorporate auditory feedback to refine articulatory movements during speech production (Tourville et al., 2008), as demonstrated by the DIVA (Guenther et al., 1995, 1998, 2006) and GODIVA models (Civier et al., 2013). These models integrate auditory input and articulatory control in speech production by starting to train the network from a babbling phase (Oller and Eilers, 1988), incorporating both feedforward and feedback pathways based on neural theories of language development. GODIVA specifically addresses sound sequence order and function in speech, complementing DIVA’s speech sound map. The dynamic interaction between action and perception in sound production emphasizes the role of auditory perception in refining the speaking process. Studies have revealed that auditory target and error maps, constructed through auditory feedback in the models, are situated in distinct regions along the posterior temporal gyrus, activated during both perception and production (Buchsbaum et al., 2001; Hickok and Poeppel, 2004). However, these models have limitations, failing to account for aspects like adaptive components in preserving speech intelligibility and sensorimotor adaptation in *Lombard speech*. While these models account for the predictive aspects of speech production, including acoustic cues and somatosensory signals, uncertainties persist regarding the integration of prosodic elements such as intonation, rhythm, and amplitude modulation. Prosodic patterns contribute to predicting syllable and word boundaries in continuous speech, as demonstrated in studies involving both children (Fernald and Mazzie, 1991) and adults (Cutler et al., 1997).

The Motor Theory (MT) of speech perception posits that listeners reference their knowledge of speech production to perceive speech, relying on an internal structure mapping acoustic cues to articulatory movements (Liberman and Mattingly, 1985). However, MT lacks an explanation for the neurophysiological pathways underlying this mapping and is based on a simplified speech production system without accounting for predictive abilities during speech perception. Hickok et al. (2009) argued for an auditory theory, suggesting the motor system’s role is limited to a minor modulatory function, consistent with the “*general auditory*” view of speech perception (Stevens, 2002). They propose two networks of auditory-phonological and lexical-conceptual at different cortical levels for mapping acoustic to linguistic concepts. The “*general auditory*” view, however, overlooks the human capacity limitations in memorizing all acoustic-to-phoneme mappings (i.e., lack of an unlimited memory), which is highly variable considering *the lack of invariance problem* in speech comprehension (Browman and Goldstein, 1990; Goldstein and Fowler, 2003).

The Direct Realistic Theory (DRT) of speech perception, akin to Motor Theory (MT), connects speech perception to the production mechanism (Fowler, 1986). Contrary to the acoustic invariance theory (Stevens, 2002), DRT posits that speech is perceived through reconstructing speakers’ articulatory gestures rather than directly decoding acoustic features. In DRT, a group of neurons directly represents articulatory patterns, mapping relevant acoustic information to phonemic units. This active theory requires neural mechanisms for speech production to reconstruct vocal tract movements. Both MT and DRT claim that gestures are perceived during speech listening, involving the reconstruction of articulatory-phonetic patterns until the execution phase begins. However, neither theory provides compelling evidence for mapping acoustic cues to phonemic categories, and they lack a component for predictive coding during perception.

Articulatory phonology underscores gestures as the fundamental units for mapping articulation to the perception of lexical items (Ohala et al., 1986; Goldstein and Fowler, 2003). In this framework, phonological events result from dynamic variations in gestural patterns during articulation, such as tongue position changes. Unlike traditional models, articulatory gestures do not strictly correspond to acoustic features at the segmental or phonemic level, leading to an overlap between the onset, plateau, and offset period during phonemic unit pronunciation, addressing the lack of invariance problem (Browman and Goldstein, 1990; Goldstein and Fowler, 2003). Syllable and word formation rely on patterns of location and constriction created by articulatory movements in the vocal tract, rather than sequences of segments and phonemes in continuous speech. Perception involves reconstructing these articulatory patterns, either directly (as in the DRT) or indirectly (as in MT) mapping from acoustic to articulatory patterns. Biological evidence, such as the activation of mirror neurons in the motor cortex during speech listening, supports this mapping, but debates persist about the necessity of the connection between motor neurons and articulatory-related activations, as discussed in the MT.

Pickering and Garrod’s integrated theory of language comprehension and production posits a psycholinguistic framework where comprehension and speech production are interlinked components, and both involve predictive coding (Pickering and Garrod, 2013). This theory addresses the connection between action, action perception, and joint action in spoken word communication. Like Moore’s model (Moore, 2007), speakers construct forward models of their actions before execution, and listeners activate the same forward model of articulation. The prediction system in both parties ensures “*signal parity*,” essential for effective communication. Predictions span semantic, syntactic, and phonological levels through covert imitation and forward modeling. Listeners use this mechanism as active feedback, closely intertwining perception and comprehension. While the model accounts for dyadic communication, it lacks explanations for the neurobiological pathways of the forward model and simplifies intention reading in verbal communication to motor behavior tasks, overlooking the broader complexity of predicting interlocutors’ intentions.

## Discussion

The extensive body of research discussed in this mini review underscores the intricate link between speech perception and production, emphasizing their bidirectional nature. Notably, online monitoring of auditory feedback plays a key role in normal speech production by using a complex sensorimotor integration mechanism to adjust phonatory and articulatory movements (Tourville et al., 2008; Behroozmand and Larson, 2011; Behroozmand et al., 2022). While existing models of speech perception-production offer valuable insights into sources of the deficit in disorders of language (e.g., aphasia) and speech (e.g., stuttering, dysarthria) (Hickok et al., 2011; Chang and Guenther, 2020), they may not fully explain the impact of impaired hearing and hearing devices (cochlear implants and hearing aids) on speech production.

**Figure 1:**
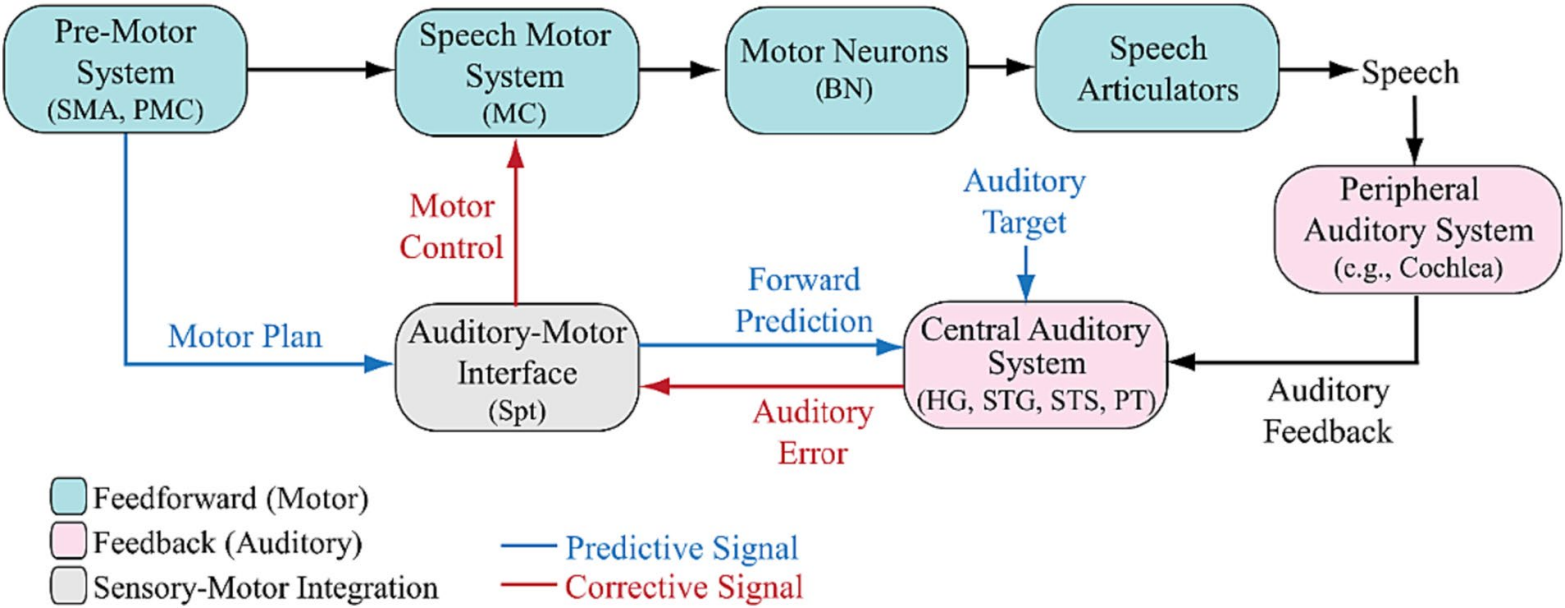
Revised auditory-motor integration model of speech with a peripheral auditory system and the relevant neurobiological pathways. In the model, an intact auditory system detects prediction errors and sensory prediction errors in response to a change in auditory feedback. The auditory-motor interface transforms speech motor plans into forward predictions of auditory feedback. The generation of corrective signals in response to errors in speech production can be disrupted due to an impaired peripheral hearing such as loss or damage to the hair cells in the cochlea in the peripheral auditory system (e.g., hearing loss or cochlear implants) or/and an impairment or distortion in the Auditory-Motor Interface (e.g., aphasia, stuttering, or dysarthria; see Hickok et al., 2011; Chang et al., 2015). HG, Heschl’s Gyrus; PT, Planum Temporale; STG, Superior Temporal Gyrus; STS, Superior Temporal Sulcus; Spt, posterior Planum Temporale; SMA, Supplementary Motor Area; PMC, Premotor Cortex; MC, Motor Cortex; BN, Brainstem Nuclei.

The integrative sensorimotor model of speech (Behroozmand et al., 2018) proposes a framework where the auditory-motor interface transforms speech motor plans into forward predictions about the auditory feedback consequences of intended productions. The original model assumes a normal auditory pathway, identifying sensory prediction errors and translating them into corrective signals through the auditory-motor interface to adjust speech motor parameters. While previous models have emphasized the role of auditory feedback system for speech, no distinction was made between the mechanisms underlying peripheral vs. central auditory processing pathways. Here, we propose a revised model that incorporates a separate module to account for the role of peripheral auditory system in speech (Peripheral Auditory System in Figure 1). This revision is a critical consideration to explicitly examine the impact of peripheral auditory dysfunction, such as in patients with hearing loss or the users of hearing assistive devices (cochlear implants and hearing aids), on speech sensorimotor processes. This model illustrates how a spectrotemporally-degraded signal, due to impaired peripheral auditory pathways, may modify components and relationships within the classical model.

We propose that these modifications impact our understanding of how peripheral auditory deficits may induce detrimental effects on the accuracy of forward predictions, the detection of errors, and the generation of corrective speech motor commands by the auditory-motor interface. In fact, impairment in the peripheral auditory system (Figure 1), such as loss or damage to cochlear mechanisms [e.g., hair cells (HCs) and auditory nerve fibers (ANs)] can create a cascade of deficiencies, impacting components of the model at different levels. Hearing impairment, particularly sensorineural hearing loss, is often linked to missing or damaged HCs in the cochlea of the inner ear (Ashmore et al., 2010; Fettiplace and Kim, 2014). This condition results in the inability of HCs to effectively transduce acoustic energy into electrical signals transmitted to the brain through ANs, leading to degraded transmission of fine- and sometimes coarse-grained spectral and temporal cues along the auditory pathway from both the left and right cochleae to the brain (Saada et al., 1996; Kral et al., 2000; Raggio and Schreiner, 2003; Middlebrooks et al., 2005; Loizou et al., 2009; Sanes and Kotak, 2011). This lack of sensory input induces neuroplastic changes in the brains of both humans (Huttenlocher and Dabholkar, 1997; Moore and Guan, 2001; Moore and Angeles, 2002; Moore and Linthicum, 2007; Iyengar, 2012; Pundir et al., 2012) and animals (Arenberg et al., 2000; Eliades and Wang, 2005; Middlebrooks et al., 2005; Eliades and Wang, 2008; Eliades and Tsunada, 2018; Middlebrooks, 2018). The spectro-temporally degraded auditory input is expected to impact initial cortical processing of speech in superior temporal gyrus (STG; e.g., spectro-temporal analysis, region-specific response to different sound frequencies) and superior temporal sulcus (STS; e.g., phonological analysis and complex processing of speech) (Hickok et al., 2011, 2023; Oganian et al., 2023), mainly in Heschl’s Gyrus (HG) and Planum temporale (PT) (Ratnanather, 2020; Oganian et al., 2023) (Central Auditory System in Figure 1), which could also lead to a deficient formation of Auditory Target (Figure 1). These areas also project back to other brain structures via the thalamus and brainstem (Kara et al., 2006; Li et al., 2012, 2013; Hribar et al., 2014; Shiell et al., 2016; Smittenaar et al., 2016; Kumar and Mishra, 2018; Pereira-Jorge et al., 2018; Shiohama et al., 2019).

The degraded signal may also impact the formation of sensory information during learning phase (Goupell, 2015; Svirsky, 2017; Ratnanather, 2020; Arjmandi et al., 2021, 2022) and their transformation into appropriate speech motor commands in Supplementary Motor Area (SMA) and/or Premotor Cortex (PMC; Pre-Motor System in Figure 1), thus impacting the motor planning, initiation of and the temporal organization of sequences of movements involved in speech production. Such distorted internal model for sensory prediction may impact the integration of motor plans and auditory input in the Auditory-Motor Interface station (Figure 1), a process believed to involve multiple cortical regions, primarily the posterior PT (Spt) (Hickok et al., 2003, 2009; Chang et al., 2015). This may potentially result in the generation of impaired forward predictions and motor control commands. Therefore, the Speech Motor System in Figure 1 is expected to be impacted because the transformation of any mismatch between the learned motor commands and auditory feedback into compensatory gestures in motor cortex (MC) requires a normal motor plan signal as well as the faithful transmission of auditory feedback (Brown et al., 2008; Simonyan and Horwitz, 2011; Tourville and Guenther, 2013; Simonyan, 2014; Scott et al., 2020). Thus, impaired ability to detect errors complicates the generation of effective corrective speech motor commands, hindering the auditory-motor interface. Motor neurons, in turns, in the brainstem nuclei (BN) may not be able to accurately innervate muscles and control components of Speech Articulators in Figure 1 that are involved in speech production such as respiratory system, vocal folds vibration, and the movement of tongue, lips, jaw, and velopharyngeal port. Despite this potential cascade of impairments, the neurophysiological pathways that explain how these components impact sensorimotor processing due to peripheral auditory system impairment remain largely unknown. Understanding these effects can help with elucidating atypical features of speech production at the segmental and suprasegmental levels exhibited by listeners with hearing loss and those with cochlear implants such as contracted vowel space (Economou et al., 1992; Langereis et al., 1997; Schenk et al., 2003; Lane et al., 2007; Ménard et al., 2007), deviated vocal pitch (Perkell et al., 1992; Svirsky et al., 1992; Lane et al., 1995) and loudness (Plant and Oster, 1986; Perkell et al., 1992; Schenk et al., 2003; Evans and Deliyski, 2007), decreased vocal stability (Campisi et al., 2005; Hocevar-Boltezar et al., 2006; Evans and Deliyski, 2007; Dehqan and Scherer, 2011; Eskander et al., 2014; Wang et al., 2017), and increased variability in voice-onset time during consonant production (Tartter et al., 1989; Economou et al., 1992; Lane et al., 1994, 1995; Kishon-Rabin et al., 1999).

In conclusion, our understanding of the speech perception-production relationship has advanced significantly. However, it remains elusive concerning the effects of impaired hearing, specifically at the peripheral level. To address challenges presented by impaired auditory feedback, such as restricted access to spectrotemporal information, there is a need to enhance existing models. A refined model with integration of the peripheral auditory system can better explain the intricate interplay between perception and production of speech in the presence of impaired auditory feedback. Experimental data from testing such a model has the potential to lay the groundwork for developing customized diagnostic tools and personalized treatment approaches, ultimately optimizing both auditory input and speech outcomes.

## Author contributions

MA: Conceptualization, Data curation, Investigation, Project administration, Resources, Supervision, Validation, Writing – original draft, Writing – review & editing. RB: Conceptualization, Resources, Validation, Writing – review & editing, Supervision.
